# Studies on Wound Healing Activity of *Heliotropium indicum* Linn. Leaves on Rats

**DOI:** 10.5402/2011/847980

**Published:** 2011-04-12

**Authors:** G. K. Dash, P. N. Murthy

**Affiliations:** ^1^Institute of Pharmacy and Technology, Salipur, Cuttack District, Odisha 754202, India; ^2^Royal College of Pharmacy and Health Sciences, Andha Pasara Road, Berhampur, Odisha 760002, India

## Abstract

The petroleum ether, chloroform, methanol, and aqueous extracts of *Heliotropium indicum* Linn. (Family: Boraginaceae) were separately evaluated for their wound healing activity in rats using excision (normal and infected), incision, and dead space wound models. The effects of test samples on the rate of wound healing were assessed by the rate of wound closure, period of epithelialisation, wound breaking strength, weights of the granulation tissue, determination of hydroxyproline, super oxide dismutase (SOD), catalase, and histopathology of the granulation tissues. Nitrofurazone (0.2% w/w) in simple ointment I. P. was used as reference standard for the activity comparison. The results revealed significant promotion of wound healing with both methanol and aqueous extracts with more promising activity with the methanol extract compared to other extracts under study. In the wound infection model (with *S. aureus* and *P. aeruginosa*), the methanol extract showed significant healing activity similar to the reference standard nitrofurazone. Significant increase in the granulation tissue weight, increased hydroxyproline content, and increased activity of SOD and catalase level with the animals treated with methanol extract in dead space wound model further augmented the wound healing potential of *H. indicum*. The present work substantiates its validity of the folklore use.

## 1. Introduction

Wound healing is the process of repair that follows injury to the skin and other soft tissues. It involves a complex series of interactions between different cell types, cytokine mediators, and the extracellular matrix. Each phase of normal wound healing, namely, hemostasis, inflammation, proliferation and remodeling is distinct although the wound healing process is continuous, with each phase overlapping the next. Several medicinal plants have been used since time immemorial for treatment of cuts, wounds, and burns and showed promising effects. Some very common plants like *Aloe vera*, *Azadirachta indica*, *Carica papaya*, *Celosia argentea*, *Centella asiatica*, *Cinnamomum zeylanicum*, *Curcuma longa*, *Nelumbo nucifera*, *Ocimum sanctum*, *Phyllanthus emblica*, *Plumbago zeylanica*, *Pterocarpus santalinus*, *Terminalia arjuna,* and *Terminalia chebula* have been extensively reported in Ayurveda, Siddha, and Unani systems of medicines for their wound healing potential [1].

*Heliotropium indicum *Linn. (Family: Boraginaceae) is a coarse foetid herb, up to 2 ft. high, found throughout India in sunny localities, on waste lands, and anthropogenic habitats, widely considered as a weed of fields [2–4]. The plant is reported to be highly valued in the folklore medicine and is believed to be useful in treating malaria, abdominal pain, fever, dermatitis, venereal diseases, insect bites, menstrual disorder, urticaria, and sore throat [5–9]. The plant decoction is considered as diuretic and remedy for the treatment of kidney stone [10, 11]. The leaf paste is applied externally to cure rheumatism and skin infections [12, 13]. The various tribes of Phulbani district of Odisha use the leaf paste over fresh cuts and wounds and claim for its promising activity.

Several pyrolizidine alkaloids, namely, echinatine, heleurine, heliotriene, lasiocarpine, indicine, indicine N-oxide, retronicine, cynoglossine, and supinine have been reported from the plant [14–19]. 

Reports on the biological activity of the plant are many. Different extracts of *H. indicum* have been studied for possible biological activities in various animal models and reported to possess significant anti-inflammatory, diuretic, abortifacient, wound healing and antitumor activities [20–23]. The active principle responsible for the antitumor activity is reported to be indicine N-oxide [24]. The aqueous extract of the leaves is reported to possess strong uterine stimulant effect [25, 26]. Earlier reports on wound healing activity of the plant have been reported on the basis of only preliminary animal models like excision and incision wound models. The present study was undertaken to provide an in-depth study on wound healing activity of the leaves using all possible models and to provide a scientific support to its use in the folklore medicines for treating wound infection.

## 2. Materials and Methods

### 2.1. Plant Material

The plant material (leaves) were collected from the forests of Phulbani district of Odisha during November 2009 and authenticated. The leaves were washed, shade dried and pulverized to coarse powder. The powdered leaves (500 g) was successively extracted with petroleum ether (40–60°C), chloroform, methanol and water for 48 h in a soxhlet extractor. Following extraction, the liquid extracts were concentrated under vacuum to yield dry extracts. Standard methods [27, 28] were used for preliminary phytochemical screening of the different extracts to know the nature of phytoconstituents present within them.

### 2.2. Animals

Healthy Wistar albino rats (150–250 g) of either sex and of approximately the same age were used for the study. They were individually housed, maintained in clean polypropylene cages and fed with commercially pellet diet (M/s Hindustan Lever Ltd., Mumbai) and water *ad libitum*. The experimental protocols were subjected to scrutiny of Institutional Animal Ethics Committee for experimental clearance (no. 1025/C/07/CPCSEA).

### 2.3. Wound Healing Activity

The selected extracts of *H. indicum *were separately evaluated for their wound healing activity in rats using excision (normal and infected), incision and dead space wound models. The effects of test samples on the rate of wound healing were assessed by the rate of wound closure, period of epithelialisation, wound breaking strength, weights of the granulation tissue, determination of hydroxyproline, super oxide dismutase (SOD), catalase and histopathology of the granulation tissue. Nitrofurazone (0.2% w/w) in simple ointment I. P. was used as reference standard for the activity comparison. The test extracts were mixed with simple ointment I. P. (10% w/w) and used in the excision and incision models. For the dead space wound model, the methanol extract was suspended in water and used.

### 2.4. Excision Wound Model (Normal Wounds)

Animals were anesthetized prior to and during creation of the wounds, with 1 mL of intravenous ketamine hydrochloride (10 mg/kg). The rats were inflicted with excision wounds as described by Morton and Malone [29] and suggested by Kamath et al. [30]. An impression was made on the dorsal thoracic region 1 cm away from vertebral column and 5 cm away from ear on the anaesthetized rat. The dorsal fur of the animals was shaved with an electric clipper and the anticipated area of the wound to be created was outlined on the back of the animals with methylene blue using a circular stainless steel stencil. A full thickness of the excision wound of circular area of 500 mm^2^ and 2 mm depth was created along the markings using toothed forceps, scalpel and pointed scissors. Haemostasis was achieved by blotting the wound with cotton swab soaked in normal saline. The entire wound was left open [31]. All surgical procedures were performed under aseptic conditions.

The control group animals (Group I) were treated with the vehicle (simple ointment I. P.), the positive control (Group II) was applied with 0.2% w/w nitrofurazone in simple ointment I. P. Other groups of animals were treated with the following: petroleum ether, chloroform, methanol or aqueous extracts of *H. indicum* at a concentration of 10% w/w in simple ointment I. P. in a similar manner.

The wound closure rate was assessed by tracing the wound on days 1, 4, 6, 8, 11, 14, and 16 after wounding days using transparent paper and a permanent marker. The wound areas recorded were measured using graph paper [4]. The percentage of wound healing was calculated of original wound size for each animal of group on predetermined days that is, 1, 4, 6, 8, 11, 14 and 16 days post-wounding for final analysis of results. Changes in wound area were calculated, giving an indication of the rate of wound contraction [5]. The period of epithelialisation was calculated as the number of days required for falling of the dead tissue remnants without any residual raw wound. The results are tabulated in Table 2.

### 2.5. Incision Wound Model

The rats were anaesthetized prior to and during creation of the wounds, with 1 mL of intravenous ketamine hydrochloride (10 mg/kg). The dorsal fur of the animals was shaved with an electric clipper. A longitudinal paravertebral incision of 6 cm long was made through the skin and cutaneous tissue on the back as described by Ehrlich and Hunt [32]. After the incision, the parted skin was sutured 1 cm apart using a surgical thread and curved needle. The wounds were left undressed [33]. Extracts were topically applied to the wound once a day. The sutures were removed on 8th post wound day and continued the application of the extract. The wound breaking strength [34] was measured on the 10th day evening after the last application. The results are tabulated in Table 3.

The healing tissues obtained on the 10th day from all four groups of animals of the incision wound model were processed for histological study [35, 36].

### 2.6. Excision Wound Model (Infected Wounds)

 The results of the excision and incision wound models revealed that the methanol extract of *H. indicum *possesses comparatively better wound healing activity compared to other test extracts under study. Therefore, infected wound model was separately performed on the methanol extract of *H. indicum* taking *Staphylococcus aureus* and *Pseudomonas aeruginosa* as the infecting bacteria.

The methods of Abo et al. [37] and Odimegwu et al. [38] was followed. The selected rats were divided into three groups, each containing 6 animals. A round seal of 20 mm diameter was impressed on the two sides of the central trunk depilated and sterilized with ethanol. Excision wound was inflicted on the rats as described earlier. Full skin thickness was excised from the marked area to get a wound measuring about 314 mm^2^. After achieving complete haemostasis by blotting the wound with cotton swab soaked in warm saline, the wound of each animal was inoculated separately with an overnight (18 h old) *S*. *aureus * and *P. aeruginosa* cultures. The animals were placed singly in individual cages. The infected wounds on each animal of the control group were treated topically with simple ointment I. P. Other groups of animals were treated separately with one of the following: 0.2% w/w nitrofurazone or 10% w/w methanol extract of *H. indicum* in simple ointment I. P. in a similar manner.

Treatments of the infected wounds commenced on the 3rd day to allow for the establishment of the infection on the wound. The wound area was measured with a transparent graph paper on 1, 4, 6, 8, 11, 14 and 16 day. Wound contraction was calculated as a percentage of the original wound size. The results are presented in Tables 4 and 5.

### 2.7. Acute Oral Toxicity Studies

Acute oral toxicity studies of the extracts were carried out as per the OECD guidelines, draft guidelines 423 [39]. Different groups of animals each containing three female rats (180–210 g) received *H. indicum* methanol extract suspended in water separately at doses of 300, 600, and 2000 mg/kg orally by gavage. Animals were observed individually after dosing once during the first 30 minutes, periodically during the first 24 hours, with special attention given during the first 4 hours and daily thereafter, for a total of 14 days. Observations included changes in skin and fur, eyes and mucous membranes, and respiratory and behaviour pattern. A special attention was directed to observations of tremors, convulsions, salivation, diarrhea, lethargy, sleep and coma. The change in body weight, food and water intake was recorded at two days interval. 

There was no mortality or morbidity observed in animals through the 14-day period following single oral administration at all selected dose levels of the methanol extract of *H. indicum*. Morphological characteristics (fur, skin, eyes, and nose) appeared normal. No tremors, convulsion, salivation, diarrhea, lethargy, or unusual behaviours such as self mutilation walking backward and were observed; gait and posture, reactivity to handling or sensory stimuli, and grip strength were all normal. There was no significant difference in body weights between control and treatment groups.

### 2.8. Dead Space Wound Model

Dead space wounds were created by implanting two preweighed sterilized polypropylene tube (2.5 length × 0.25 cm diameter) beneath the dorsal paravertebral skin of the anaesthetized rats [40]. The animals were randomly divided into two groups of six each. The control group animals were provided with plain drinking water, and the other group rats were separately administered with the methanol extract of *H. indicum* at a dose of 100 mg/kg daily. On the 10th post-wounding day, the granulation tissue formed on the implanted tubes was carefully detached from surfaces of the tubes. The wet weight of the granulation tissue collected was noted. The tissue samples were dried at 60°C for 12 h and weighed to determine the dry granulation tissue weight. The results are depicted in Table 6. 

The dried tissue (50 mg) was added to 1 mL 6 M HCl and kept at 110°C for 24 h. The neutralized acid hydrolysate of the dry tissue was used for the determination of hydroxyproline [41]. Part of the granulation tissue was collected in phosphate-buffered saline for the estimation of antioxidant enzymes superoxide dismutase (SOD) [42] and catalase [43].

### 2.9. Histological Studies

**Figure 1:**
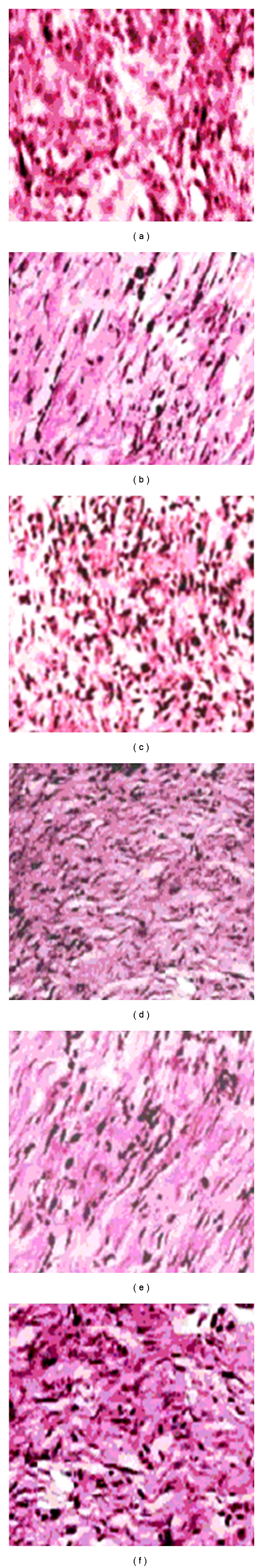
Histology of granulation tissue of *H. indicum. * (a) Granulation tissue of Group I animal (control) showing with less collagen and more macrophages. (b) Granulation tissue of Group II (standard) animal showing significant collagenation, lesser fibroblasts and capillaries. (c) Section of granulation tissue of group III animal (pet-ether extract of *H. indicum*) showing with less collagenation with less monocytes, fibroblasts and capillaries. (d) Granulation tissue of Group IV (chloroform extract of *H. indicum*) animal showing with moderate collagen and moderate macrophages. (e) Histological section of granulation tissue of Group V animal treated with methanol extract of *H. indicum* showing significant increased collagenation, few macrophages and capillaries. (f) Section of granulation tissue of Group VI (aqueous extract of *H. indicum*) animal showing moderate collagenation with less macrophages, fibroblasts, and capillaries.

For histological studies, pieces of granulation tissues from incision wound model were fixed in 10% neutral formalin solution for 24 h and dehydrated with a sequence of ethanol-xylene series of solutions. The materials were filtered and embedded with paraffin (40–60°C). Microtome sections were taken at 10 *μ* thickness. The sections were processed in alcohol-xylene series and stained with hemotoxylin-eosin dye. The histological changes were observed under a microscope. Photographs were taken from each slide and presented in Figure 1.

### 2.10. Statistical Analysis

The data obtained in the studies were subjected to one way of analysis of variance (ANOVA) for determining the significant difference. The inter group significance was analyzed using Dunnet's *t*-test. A *P*  value < .05 was considered to be significant. All the values were expressed as Mean ± SEM.

## 3. Results

The preliminary phytochemical screening of *H. indicum* leaf extracts showed presence of steroids and sterols, triterpenoids, alkaloids, flavonoids, saponins, tannins and phenolic substances, gums and mucilages, carbohydrates, and proteins, respectively, in different extracts (Table 1).

The results of wound healing effects of *H. indicum* showed significant promotion of wound healing activity with both aqueous and methanol extracts in the excision and incision wound models. In excision wound model, the mean percentage closure of wound area was calculated on the 4, 6, 8, 11, 14 and 16 post-wounding days as shown in Table 2. The methanol extract-treated animals showed faster epithelialisation of wound than the animals treated with aqueous leaf extract. The percentage of wound closure was 100 ± 00 in the case of standard drug nitrofurazone on 14th day of treatment, where as the methanol extract demonstrated similar effects on 16th day. But the petroleum ether and chloroform extracts did not reveal significant activity. The period of epithelialization was 16.23 ± 0.98 days for the methanol extract treated group of animals as against 13.5 ± 1.54 for the standards drug-treated group.

In incision wound model (Table 3), the methanol and aqueous extract treated animals showed significant increase in breaking strength (478.55 ± 12.63 and 378.63 ± 18.02 resp.), when compared to the control (327.5 ± 16.58). The mean breaking strength was also significant in animals treated with standard drug nitrofurazone (491.21 ± 16.26) whereas the other extracts failed to produce significant effects. The methanol extract showed better activity (*P* < .01) than the aqueous extract (*P* < .05). 

The results of the excision and incision wound models revealed that the methanol extracts of* H. indicum* possess better wound healing activity compared to other test extracts under study, so the work was further extended to investigate its activity on infection of wounds model. In the wound infection model (with *S. aureus*), the methanol extract showed significant activity (*P* < .01) from 6th day and throughout the experiment similar to the reference standard nitrofurazone (Table 4). But the wounds infected with *P. aeruginosa*, significant activity was observed from 8th day (Table 5).

In dead space wound model (Table 6), the methanol extract-treated animals showed significant increase in dry weight of granulation tissue. Estimation of hydroxyproline content in the granulation tissue revealed that the animal groups treated with methanol extract had high hydroxyproline content (7085.82 ± 687.47) as against the control group (2933.33 ± 326.60). In case of the antioxidant parameters, rats treated with the methanol extracts of *H. indicum* showed significant increase in the activity of SOD and catalase level in granulation tissue compared with controls. 

The histological profiles of granulation tissue of control animals (Figure 1(a)) showed more macrophages and less collagenation. The sections of granulation tissue of methanol and aqueous extract-treated animals showed the sign of tissue repair with increased collagen formation (Figures 1(e) and 1(f)) and less macrophages. Whereas, in the petroleum ether and chloroform extract-treated animals the healing activity was comparatively lesser with moderate collagenation and retention of the macrophages (Figures 1(c) and 1(d)). The nitrofurazone treated group of animals also revealed increased collagen fibers with few macrophages (Figure 1(b)).

## 4. Discussion

The results of the present study revealed that animals treated with methanol and aqueous extracts of *H. indicum* showed faster rate of epithelialization in excision wound model compared to other extracts under study. The chloroform extract of the selected plants also produced promising results, but the effects are seen to be of lesser extent than the corresponding methanol and aqueous extracts. The petroleum ether extract of all the plant materials did not produce significant results. The wound healing effects of the chloroform, methanol, and aqueous extracts may be attributed to the presence of phytoconstituents like alkaloids, triterpenoids, tannins and flavonoids in the extracts which are known to promote the wound healing process mainly due to their antimicrobial property. Flavonoids and triterpenoids are also known to promote the wound healing process mainly due to their astringent and antimicrobial property, which seems to be responsible for wound contraction and increased rate of epithelialisation [44–46]. In the present laboratory all the surgical interventions were carried out under sterile conditions and animals were closely observed for any infection; those which showed signs of infection were separated and excluded from the study. This is very important and researchers proved that the control microbial infection is necessary for better wound healing and its management [47, 48].

Increase in skin breaking strength and tissue breaking strength in incision and dead space wound model respectively indicated enhanced collagen maturation. Increase in the granulation tissue dry weight and hydroxyproline content indicated the high collagen turnover which may be due to the activity of some phytoconstituents like flavonoids which are known to reduce lipid peroxidation not only by preventing or slowing the onset of cell necrosis but also by improving vascularity. Hence, any drug that inhibits lipid peroxidation is believed to increase the viability of collagen fibrils by increasing the strength of collagen fibers, by increasing the circulation, by preventing the cell damage and by promoting the DNA synthesis [49]. Hence, the wound healing promoting activity of *H. indicum *may also be attributed to the antioxidant and antibacterial potency of the active constituents present in them. 

Thus, wound healing property of the methanol and aqueous extracts may be attributed to the phytoconstituents they contain, which may be either due to their individual or additive effect that fastens the process of wound healing. The methanol extracts of each selected plant materials were found to possess better wound healing property over other extracts. At this stage, it is difficult to say which component(s) of the extracts are responsible for the wound healing activity. However, further phytochemical studies are needed to isolate the active compound(s) responsible for these pharmacological activities.

## Figures and Tables

**Table 1:** Preliminary phytochemical screening of different extracts of *H. indicum *leaves.

Extract	Alkaloids	Carbohydrates	Glycosides	Gums and mucilages	Proteins and amino acids	Tannins and phenolic compounds	Steroids and sterols	Triterpenoids	Saponins	Flavonoids
Pet. Ether	−	−	−	−	−	−	+	+	−	−
Chloroform	+	−	−	−	−	−	+	+	−	−
Methanol	+	−	−	−	+	+	−	−	+	+
Aqueous	−	+	−	+	+	+	−	−	+	+

(+): Present; (−): Absent.

**Table 2:** Effect of various extracts of *H. indicum* leaves on percentage (%) wound closure (excision wound model).

Group	Treatment	Percentage (%) wound closure						Period of epithelialization (No. of days)
		4th days	6th days	8th days	11th days	14th days	16th days	
I	Control	23.52 ± 1.21	37.72 ± 1.58	51.92 ± 1.71	71.28 ± 2.23	79.24 ± 1.18	83.56 ± 1.03	23.16 ± 0.71
II	Nitrofurazone (0.2% w/w)	48.53 ± 2.87*	74.23 ± 3.32**	84.8 ± 1.26**	96.54 ± 1.29**	100 ± 00**	—	13.5 ± 1.54**
III	Pet ether extract	22.15 ± 1.75	38.73 ± 2.16	44.70 ± 3.25	70.58 ± 1.88	79.69 ± 1.22	83.88 ± 2.01	20.5 ± 1.33
IV	Chloroform extract	24.19 ± 1.03	44.41 ± 1.22	61.88 ± 1.83	75.68 ± 2.31	81.26 ± 1.65	85.80 ± 1.43	19. ± 0.91*
V	Methanol extract	28.88 ± 1.95	48.34 ± 1.86*	79.78 ± 1.91*	86.37 ± 1.01**	97.76 ± 2.22**	100 ± 00**	16.23 ± 0.98**
VI	Aqueous Extract	29.23 ± 1.65	48.01 ± 2.01*	65.28 ± 2.83*	77.25 ± 1.11*	90.42 ± 1.71**	93.7 ± 1.22	18.5 ± 0.77*

Values are expressed as mean ± S.E. (*n* = 6). All columns are significant using ANOVA.

*P < .05, **P < .01 when compared to control; Dunnet's *t*-test.

**Table 3:** Effect of various extracts of *H. indicum *leaves on wound breaking strength (incision wound model).

Group	Treatment	Breaking strength (g)
I	Control	327.5 ± 16.58
II	Nitrofurazone (0.2% w/w)	491.21 ± 16.26**
III	Pet ether extract	336.23 ± 15.64
IV	Chloroform extract	347 ± 14.63
V	Methanol extract	478.55 ± 12.63**
VI	Aqueous extract	378.63 ± 18.02*

Values are expressed as mean ± S.E. (*n* = 6). All columns are significant using ANOVA.

*P < .05, **P < .01 when compared to control; Dunnet's *t*-test.

**Table 4:** Screening for wound healing activity of the methanol extracts of the selected plants (excision wound model) by inoculated with* Stapphylococcus aureus. *

Group	Treatment	Percentage (%) wound closure						Period of epithelialization (no. of days)
		4th days	6th days	8th days	11th days	14th days	16th days	
I	Control	10 ± 1.29	19.5 ± 2.34	34 ± 3.83	44.5 ± 5.75	52 ± 4.53	58.4 ± 5.85	29.12 ± 2.65
II	Nitrofurazone (0.2% w/w)	15.83 ± 6.21	36.33 ± 2.98**	59.5 ± 4.48**	76.33 ± 4.91**	90.5 ± 3.39**	97.33 ± 1.97**	18.03 ± 1.23**
III	Methanol extract (HI)	15.5 ± 1.4	33.5 ± 3.6**	57.66 ± 3.84**	63.16 ± 3.89**	77.66 ± 3.71**	93 ± 2.25**	19.11 ± 0.88**

Values are expressed as mean ± S.E. (*n* = 6). All columns are significant using ANOVA.

*P < .05, **P < .01 when compared to control; Dunnet's *t*-test. (HI-H. indicum).

**Table 5:** Screening for wound healing activity of the methanol extract of the selected plants (excision wound model) by inoculated with* P. aeruginosa. *

Group	Treatment	Percentage (%) wound closure						Period of epithelialization (no. of days)
		4th days	6th days	8th days	11th days	14th days	16th days	
I	Control	8.22 ± 1.81	15.6 ± 1.22	21.08 ± 2.71	32.41 ± 3.33	46.61 ± 3.85	51.13 ± 2.15	30.33 ± 2.23
II	Nitrofurazone (0.2% w/w)	14.21 ± 1.09	25.73 ± 3.81*	39.43 ± 2.22**	57.63 ± 3.11**	78.56 ± 2.48**	92.53 ± 3.25**	18.73 ± 1.81**
III	Methanol extract (HI)	12.5 ± 1.25	21.5 ± 2.81	32.26 ± 2.14*	49.6 ± 2.18**	70.22 ± 3.81**	84.26 ± 2.55**	20.10 ± 0.48*

Values are expressed as mean ± S.E. (*n* = 6). All columns are significant using ANOVA.

*P < .05, **P < .01 when compared to control; Dunnet's *t*-test. (HI-H. indicum).

**Table 6:** Wound healing effects of the methanol extracts of* H. indicum* in dead space wound model, hydroxyproline content in granulation tissues and the level of antioxidant enzymes in granuloma tissue.

Treatment	Wet tissue weight (mg)	Dry tissue weight (mg)	Concentration of hydroxyproline (mg/100 g dry tissue)	Superoxide dismutase (units/mg)	Catalase (unit/mg)
Control	91.36 ± 2.37	42.22 ± 2.07	2933.33 ± 326.60	0.117 ± 0.011	0.08 ± 0.013
Methanol extract (HI)	115.26 ± 3.69**	66.0 ± 5.01**	7085.82 ± 687.47**	0.175 ± 0.018*	0.48 ± 0.03*

Values are expressed as mean ± S.E. (*n* = 6).

*P < .05, **P < .01 when compared to control; Dunnet's *t*-test. (HI-H. indicum).

## References

[1] Kumar B, Vijayakumar M, Govindarajan R, Pushpangadan P (2007). Ethnopharmacological approaches to wound healing-Exploring medicinal plants of India. *Journal of Ethnopharmacology*.

[2] Chadha YR (1985). *The Wealth of India*.

[3] Kirtikar KR, Basu BD (1994). *Indian Medicinal Plants: Bishen Singh Mahendrapal Singh*.

[4] Stewart R (1997). Herbalism: most common form of medicine available. *The Eastern Pharmacist*.

[5] Muthu C, Ayyanar M, Raja N, Ignacimuthu S (2006). Medicinal plants used by traditional healers in Kancheepuram District of Tamil Nadu, India. *Journal of Ethnobiology and Ethnomedicine*.

[6] Togola A, Diallo D, Dembélé S, Barsett H, Paulsen BS (2005). Ethnopharmacological survey of different uses of seven medicinal plants from Mali, (West Africa) in the regions Doila, Kolokani and Siby. *Journal of Ethnobiology and Ethnomedicine*.

[7] Asprey GF, Thornton P (1955). Medicinal plants of Jamaica. *The West Indian Medical Journal*.

[8] Giron LM, Freire V, Alonzo A, Caceres A (1991). Ethnobotanical survey of the medicinal flora used by the Caribs of Guatemala. *Journal of Ethnopharmacology*.

[9] Duttagupta S, Dutta PC (1977). Pharmacognostic study of the leaf of *Heliotropium indicum*. *Journal of Crude Drug Research*.

[10] Quisumbing E (1951). Medicinal Plants of Phillipines.

[11] Berhault J (1974). *Floore Illustree du Senegal. Govt. Senegal, Min Rural Development*.

[12] Nagaraju N, Rao KN (1990). A survey of plant crude drugs of Rayalaseema, Andhra Pradesh, India. *Journal of Ethnopharmacology*.

[13] Barrett B (1994). Medicinal plants of Nicaragua’s Atlantic Coast. *Economic Botany*.

[14] Misawa M, Hayashi M, Takayama S (1983). Production of antineoplastic agents by plant tissue cultures. I. Induction of callus tissues and detection of the agents in cultured cells. *Planta Medica*.

[15] Hoque MS, Ghani A, Rashid H (1976). Alkaloids of *Heliotropium indicum* Linn. Grown in Bangladesh. *Bangladesh Pharmaceutical Journal*.

[16] Medina FR, Woodbury R (1979). Terrestrial plants Molluscicial to Lymnacid host of Fascilisis hepatica in Puertorico. *Journal of Agriculture of the University of Puerto Rico*.

[17] Willaman JJ, Li H (1968). Alkaloid bearing plants and their contents alkaloids. *Loydia*.

[18] Birecka H, Dinolfo TE, Martin WB, Frohlich MW (1984). Polyamines and leaf senescence in pyrrolizidine alkaloid-bearing Heliotropium plants. *Phytochemistry*.

[19] Duttagupta S, Dutta PC (1998). Pharmacognostic study of the leaf of *H. indicum*. *Journal of Crude Drug Research*.

[20] Asprey GF, Thornton P (1955). Medicinal plants of Jamaica. *The West Indian Medical Journal*.

[21] Ogunbinu AO, Flamini G, Cioni PL, Adebayo MA, Ogunwande IA (2009). Constituents of *Cajanus cajan* (L.) Millsp., *Moringa oleifera* Lam., *Heliotropium indicum* L. and *Bidens pilosa* L. from Nigeria. *Natural Product Communications*.

[22] Andhiwal CK, Has C, Vershney RP (1981). Chemical and pharmacological studies of *H. indicum*. *Indian Drugs*.

[23] Kugelman M, Liu WC, Axelrod M, McBride TJ, Rao KV (1976). Indicine-N-oxide: the antitumor principle of *Heliotropium indicum*. *Lloydia*.

[24] Srinivas K, Rao MEB, Rao SS (2000). Anti-inflammatory activity of *Heliotropium indicum* linn. and *Leucas aspera* spreng. in albino rats. *Indian Journal of Pharmacology*.

[25] Barros GSG, Matos FJA, Vieira JEV, Sousa MP, Medeiros MC (1970). Pharmacological screening of some Brazillian plants. *Journal of Pharmacy and Pharmacology*.

[26] Vieira JEV, Barros GSG, Medeiros MC, Matos FGA, Souza MP (1968). Pharmacological screening of plants of North East Brazil. *Revista Brasileira de Ciências Farmacêuticas*.

[27] Harborne JB (1984). *Phytochemical Methods: A Guide to Modern Techniques of Plant Analysis*.

[28] Wagner H, Bladt S (1996). *Plant Drug Analysis*.

[29] Morton JJ, Malone MH (1972). Evaluation of vulneray activity by an open wound procedure in rats. *Archives Internationales de Pharmacodynamie et de Therapie*.

[30] Kamath JV, Rana AC, Chowdhury AR (2003). Pro-healing effect of *Cinnamomum zeylanicum* bark. *Phytotherapy Research*.

[31] Lowry OH, Rosenbrough NJ, Farr AL, Randall RJ (1951). Protein measurement with the Folin phenol reagent. *The Journal of Biological Chemistry*.

[32] Ehrlich HP, Hunt TK (1969). The effects of cortisone and anabolic steroids on the tensile strength of healing wounds. *Annals of Surgery*.

[33] Lee KH (1968). Studies on the mechanism of action of salicylates. 3. Effect of vitamin A on the wound healing retardation action of aspirin. *Journal of Pharmaceutical Sciences*.

[34] Reddy JS, Rao PR, Reddy MS (2002). Wound healing effects of *Heliotropium indicum, Plumbago zeylanicum* and *Acalypha indica* in rats. *Journal of Ethnopharmacology*.

[35] Nayak S, Nalabothu P, Sandiford S, Bhogadi V, Adogwa A (2006). Evaluation of wound healing activity of *Allamanda cathartica.* L. and *Laurus nobilis.* L. extracts on rats. *BMC Complementary and Alternative Medicine*.

[36] Umachigi SP, Jayaveera KN, Ashok Kumar CK (2008). Studies on Wound Healing Properties of *Quercus infectoria*. *Tropical Journal of Pharmaceutical Research*.

[37] Abo A, Olugbuyiro JAO, Famakinde SA (2004). Anti-infective and Wound healing properties of *Flabellaria paniculata*. *African Journal of Biomedical Research*.

[38] Odimegwu DC, Ibezim EC, Esimone CO, Nworu CS, Okoye FBC (2008). Wound healing and antibacterial activities of the extract of *Dissotis theifolia* (Melastomataceae) stem formulated in a simple ointment base. *Journal of Medicinal Plants Research*.

[39] Anonymous

[40] Ilango K, Chitra V (2010). Wound healing and anti-oxidant activities of the fruit pulp of *Limonia acidissima* linn (Rutaceae) in rats. *Tropical Journal of Pharmaceutical Research*.

[41] Neuman RE, Logan MA (1950). The determination of hydroxyproline. *The Journal of Biological Chemistry*.

[42] Misra HP, Fridovich I (1972). The role of superoxide anion in the autoxidation of epinephrine and a simple assay for superoxide dismutase. *The Journal of Biological Chemistry*.

[43] Aebi HE (1974). *Methods of Enzymatic Analysis*.

[44] Ya C, Gaffney SH, Lilley TH, Haslam E (1988). *Chemistry and Significance of Condensed Tannins*.

[45] Tsuchiya H, Sato M, Miyazaki T (1996). Comparative study on the antibacterial activity of phytochemical flavanones against methicillin-resistant *Staphylococcus aureus*. *Journal of Ethnopharmacology*.

[46] Scortichini M, Rossi MP (1991). Preliminary in vitro evaluation of the antimicrobial activity of terpenes and terpenoids towards *Erwinia amylovora* (Burrill). *Journal of Applied Bacteriology*.

[47] Levine S (1970). The effect of povidone-iodine in controlling skin flora beneath occlusive dressings. *Journal of the American Podiatry Association*.

[48] Muhammad HS, Muhammad S (2005). The use of *Lawsonia inermis* linn. (*henna*) in the management of burn wound infections. *African Journal of Biotechnology*.

[49] Getie M, Gebre-Mariam T, Rietz R, Neubert RHH (2002). Evaluation of the release profiles of flavonoids from topical formulations of the crude extract of the leaves of *Dodonea viscosa* (Sapindaceae). *Pharmazie*.

